# Synergistic Effect of Polyphenol-Rich Complex of Plant and Green Propolis Extracts with Antibiotics against Respiratory Infections Causing Bacteria

**DOI:** 10.3390/antibiotics11020160

**Published:** 2022-01-26

**Authors:** Anna Ramata-Stunda, Zaiga Petriņa, Valda Valkovska, Mārtiņs Borodušķis, Līga Gibnere, Eleonora Gurkovska, Vizma Nikolajeva

**Affiliations:** 1 Department of Microbiology and Biotechnology, Faculty of Biology, University of Latvia, 1 Jelgavas Str., LV-1004 Riga, Latvia; martins.boroduskis@lu.lv; 2 Microbial Strain Collection of Latvia, University of Latvia, 1 Jelgavas Str., LV-1004 Riga, Latvia; zaiga.petrina@lu.lv; 3 Faculty of Chemistry, University of Latvia, 1 Jelgavas Str., LV-1004 Riga, Latvia; valda.valkovska@lu.lv; 4 Silvanols Ltd., 2 Kurbada Str., LV-1009 Riga, Latvia; liga.gibnere@silvanols.lv (L.G.); eleonora.gurkovska@silvanols.lv (E.G.)

**Keywords:** polyphenols, synergy, respiratory infections, green propolis, *Olea europea* leaf extract, *Tabebuia avellanedae* bark

## Abstract

Bacterial infections are a prevalent complication after primary viral respiratory infections and are associated with high morbidity and mortality. Antibiotics are widely used against bacterial respiratory pathogens; however, the rise in antibiotic-resistant strains urges us to search for new antimicrobial compounds, including ones that act synergistically with antibiotics. In this study, the minimal inhibitory (MIC) and minimal bactericidal (MBC) concentrations of a polyphenol-rich complex of green propolis, *Tabebuia avellanedae* bark, and *Olea europaea* leaf extracts against *Staphylococcus aureus*, *Haemophilus influenzae,* and *Klebsiella pneumoniae* were determined, followed by an analysis of the synergistic effect with clarithromycin, azithromycin, and amoxiclav (875/125 mg amoxicillin/clavulanic acid). A combination of extracts showed activity against all three bacterial strains, with MIC values ranging from 0.78 to 12.5 mg/mL and MBC values from 1.56 to 12.5 mg/mL. The extracts showed synergistic activity with azithromycin and clarithromycin against *S. aureus*, with clarithromycin against *K. pneumoniae,* and with all three tested antibiotics against *H. influenzae*. Synergy with clarithromycin was additionally evaluated in a time-kill assay where the synergistic effects against *S. aureus* and *K. pneumoniae* were seen within the first 6 h of incubation. The results show the potential of polyphenol-rich extracts in enhancing the efficacy of antibiotic therapy and indicate their potential to be used in the management of respiratory infections.

## 1. Introduction

Respiratory tract infections are extremely prevalent, being among the most diagnosed diseases in primary and secondary care. Bacterial infections, especially pneumonia, are a common complication after primary infection with respiratory viruses such as influenza viruses, rhinoviruses, and coronaviruses and are often characterized by severe disease and high mortality. Common bacterial pathogens associated with respiratory tract infections are *Haemophilus influenzae*, *Streptococcus pneumoniae*, *Branhamella (Moraxella) catarrhalis*, *Staphylococcus aureus*, *Streptococcus pyogenes*, *Klebsiella pneumoniae,* and *Pseudomonas aeruginosa*. Clinical challenges of these infections associated with increased rates of antimicrobial resistance are observed among these pathogens [1,2,3].

Antibiotics are widely used in the treatment of respiratory tract infections. The preferred treatment for bacterial infections is generally broad-spectrum antibiotics, but this can result in undesirable side effects that have a negative impact on the normal host microflora. To avoid disease progression, complications, and negative outcomes, alternative approaches to eliminate bacterial pathogens are necessary [1,2]. Although different groups of antibiotics with different mechanisms of action are available, increasing rates of antibiotic resistance limit their use and efficacy. Inappropriate prescription and use of antibiotics, along with a lack of new effective antimicrobials, make the situation even worse [4,5]. To confront multidrug-resistant pathogens as well as the emergence of new strains, a re-evaluation of the choice and need for antibiotics, and the search for new antimicrobial compounds are needed [6,7].

Plants are valuable sources of antimicrobial compounds. There are over 1340 plants with known antimicrobial activity, and over 30,000 antimicrobial plant-derived compounds have been characterized [8]. Current studies and applications include both purified forms of specific plant metabolites as well as crude extracts. Unlike antibiotics, bacteria rarely develop resistance to plant products [9]. Another advantage is the lack of adverse effects characteristic of conventional antibiotics [10,11,12]. Particularly interesting are phenolic compounds, one of the most diverse groups of secondary plant metabolites with various biological functions, including antibacterial, antiviral, antifungal, antioxidative, and anti-inflammatory activities. Phenolic compounds act on the bacterial cell membrane, interfere with nucleic acid synthesis, inhibit bacterial metabolism, coagulate cytoplasmic proteins, and interfere with biofilm formation. Apart from direct antimicrobial activity, plant secondary metabolites have indirect activities, such as stimulation of the host’s immune response and modification of resistance mechanisms. The activity of extracts or isolated compounds varies due to the chemical composition and structures of the compounds [8,13,14]. Several plant-derived antimicrobials are synergistic with antibiotics; thus, they can be combined with standard antibiotic therapy for enhanced efficacy. The potential of plant compounds to substitute antibiotics in the case of resistant strains has attracted interest and has been investigated [13,15,16,17,18,19,20].

GoImmune Strong^®^ is a registered food supplement with antioxidative activity and is recommended for use in the cold season to promote immunity. The product contains a standardized mixture of Brazilian green propolis extract, olive (*Olea europea*) leaf extract, and tabebuia (*Tabebuia avellanedae)* bark extract. The composition was developed based on data in scientific literature. After the launch, follow-up of product use pointed to the potential efficacy for prevention of respiratory infections and strengthening immunity during an illness. These observations raised a practical question about the concomitant use of this herbal product and different antibiotics in case of bacterial infection.

*O.europea* is well known for its antibacterial and antifungal activity, and substances, including oleuropein, oleanolic acid, hydroxytyrosol, and tyrosol, have been identified as the compounds that provide this activity. Different parts of the olive are used; however, leaves have been identified as the best source of antimicrobial substances. Although purified olive leaf compounds have shown noteworthy activity, it has been reported that crude extracts are more effective, indicating the synergistic activities among the individual constituents of *O. europaea* extracts [21,22,23,24,25,26]. Reports on the spectrum of olive leaf antimicrobial activity are contradictory. In some studies, activity against a few microorganisms was detected [22,26], while others report broad activity against both Gram-negative and Gram-positive bacteria, including respiratory pathogens *S. aureus* and *K. pneumoniae* [27]. The synergy between antibiotics and olive leaf extracts has been reported. The combination of ampicillin with polyphenol-rich olive leaf extract or individual biophenols resulted in a better antibacterial effect against *S. aureus* and *Escherichia coli* than ampicillin alone [28].

Propolis, another component of the GoImmune Strong^®^ complex, is a resinous substance that honeybees (*Apis mellifera*) and stingless bees (*Melipona mondury*, *M. scutellaris)* produce by mixing their salivary gland excretions with exudate from the buds, leaves, stems, branches, and bark of plants. The chemical composition of propolis depends on its geographical origin, local flora, the species of bee, and the season [29]. The antimicrobial effects of propolis against various bacteria, yeasts, and viruses are well documented. These properties have been attributed to phenolic compounds [30,31,32,33,34,35,36,37,38]. Propolis extracts exert their antibacterial potential using two distinct mechanisms: either by promoting host immune responses or via direct interaction with bacterial cells. Efficacy against Gram-positive bacteria dominates, with Gram-negative bacteria being less susceptible. Activity against *S. aureus* is most frequently reported [39,40,41,42]. The potent bacteriostatic and bactericidal effects of propolis can be explained by its combined action on the inhibition of protein synthesis, bacterial growth, and cell lysis [42]. A combination of propolis and antibiotics to enhance the efficacy of antibacterial therapy appears promising. Propolis exerts synergistic effects with antibiotics acting on the bacterial wall synthesis and ribosome function, but it does not seem to interact with antibiotics acting on nucleic acids or folic acid biosynthesis [42,43,44,45,46,47,48]. Synergy with antibiotics against respiratory pathogens has been shown [49,50]. Different types of propolis are used in the food and pharmaceutical industries. Brazilian green propolis is derived from apical buds and young leaves of *Baccharis dracunculifolia.* This is one of the most studied types of propolis, with antibacterial activity against Gram-positive bacteria reported as a characteristic feature [50,51,52,53].

Plants from the genus *Tabebuia* are used in traditional medicine in Latin American countries and are known for their anti-inflammatory, antimicrobial, and anticancer activities. *Tabebuia avellanedae* bark extracts inhibit the growth of Gram-positive bacteria. The antimicrobial effect is attributed to the phenolic compound lapachol and other naphthoquinones that are the main components of *Tabebuia* spp. extracts. Limited data are available about the mechanism of antimicrobial activity and its interaction with other antimicrobial substances. It is supposed that phenolic compounds of *Tabebuia* induce oxidative stress in the bacterial cell membrane and interfere with ATP synthesis [54,55,56,57].

The accumulated evidence from various published studies on the antibacterial activity of individual components of the GoImmune Strong^®^ complex and the growing need for improved strategies to tackle bacterial infections encouraged us to investigate the in vitro efficacy of the complex against respiratory pathogens and to evaluate its synergy with selected antibiotics, with the aim to repurpose the complex for prophylaxis and therapy of respiratory infections.

## 2. Results

### 2.1. Total Content of Polyphenols

The total phenolic content of individual extracts as well as the GoImmune Strong^®^ complex was determined using Folin–Ciocalteu assay (Table 1). *O.europaea* extract had the highest concentration of total polyphenols; however, a high content was detected in the other two extracts as well. The polyphenol concentration in the combination of the extracts was 63.37 mg/g DW.

### 2.2. Chemical Composition

A chromatographic analysis confirmed the presence of the characteristic compounds in all three extracts. A total of 37 compounds, including 10 unidentified, were found in olive leaf extract (Table 2.). Oleuropein, hydroxytyrosol, and verbascoside were the dominating compounds. A total of 48 compounds were identified in *Tabebuia avellanedae* bark extract, with hydroxybenzoic acid, verbascoside, isoverbascoside, with derivatives of rutin and quercitin being the dominating ones (Table 3). Chromatographic analysis of green propolis extract revealed the high concentrations and diversity of flavonoids, a total of 51 compounds were found. Among dominating compounds, p-coumarinic acid, coumaric acid prenylesters, dicaffeoylquinic acid isomers, and 4-hydroxy-3-prenylcinnamic acid were identified (Table 4). Results show that all three extracts are rich in phenolic compounds, including those that have been characterized before for their antimicrobial activity. The content of oeleuropein was lower than that claimed by the manufacturer of the olive leaf extract, and analysis did not confirm the presence of lapachol in tabebuia bark extract. These differences might be due to the variations in sample preparation and analytical methods used.

### 2.3. Antibacterial Activity

Antimicrobial activity assay revealed the activity of the complex against *S. aureus, H. influenzae, and K. pneumoniae (*Table 5). The complex was active against all three bacterial strains with MIC ranging from 0.78 mg/mL in the case of *S. aureus* to 12.5 mg/mL in the case of *K. pneumoniae*. MBC values ranged from 1.56 mg/mL for *S. aureus* to 12.5 mg/mL for *H. influenzae* and *K. pneumoniae*.

### 2.4. Combined Effect of GoImmune Strong^®^ Complex and Antibiotics

#### 2.4.1. Determination of Fractional Inhibitory Concentrations (FIC) and FIC Index (FICI)

Checkerboard dilutions were used to test the interaction of GoImmune Strong^®^–antibiotic combinations. Fractional inhibitory concentrations and FIC indexes were calculated to evaluate the synergy of the complex with azithromycin, clarithromycin, and amoxiclav. Three different methods were used to interpret the results (Table 6). Synergy with all tested antibiotics against *H. influenzae* was detected. The complex was synergic against *S. aureus* with azithromycin and clarithromycin, whereas the combination with clarithromycin had the strongest inhibitory effect. For *K. pneumoniae,* only synergy with clarithromycin, according to Fratini et al.’s 2017 interpretation method, was detected.

#### 2.4.2. Time-Kill Assay

**Figure 1:**
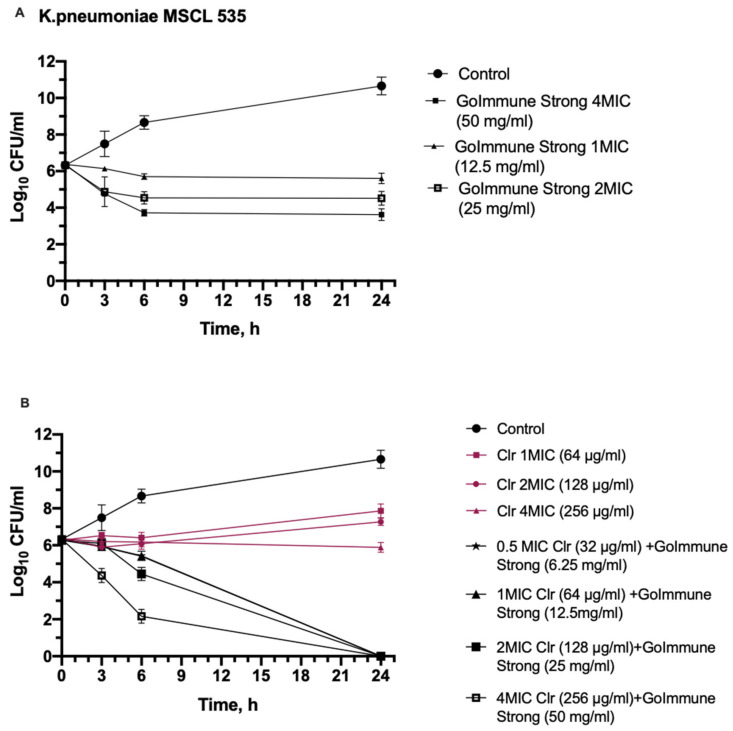
Time-kill curves show synergistic interaction between GoImmune Strong^®^ complex and clarithromycin (Clr) against *K. pneumoniae*. (**A**)—time-kill curves of GoImmune Strong^®^ complex at different concentrations. (**B**)—Time-kill curves of combination of GoImmune Strong^®^ complex and clarithromycin. Each time-curve experiment was performed in duplicate and diluted samples were plated in duplicate on agar plates. Error bars indicate standard deviations.

**Figure 2:**
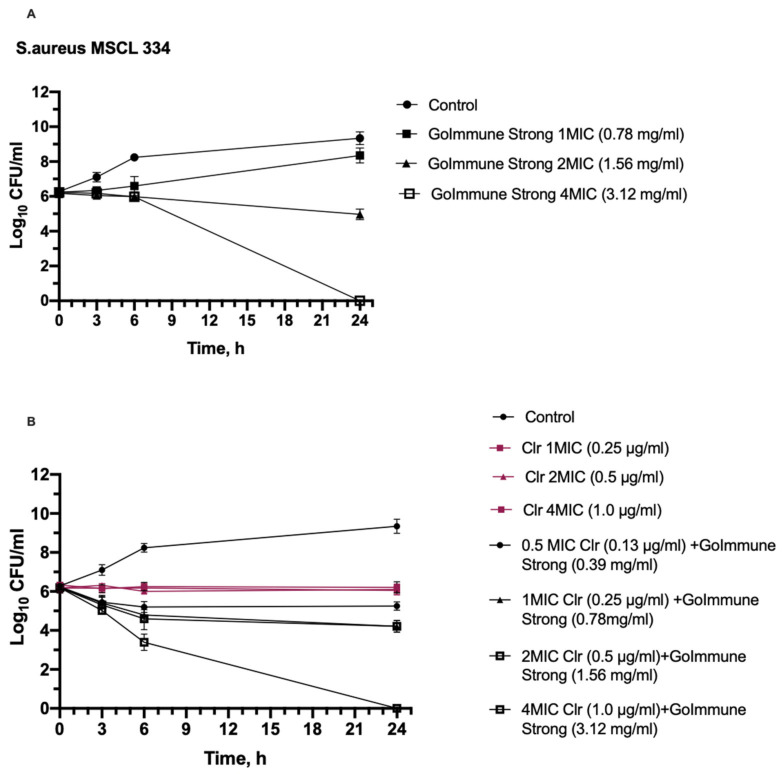
Time-kill curves show synergistic interaction between GoImmune Strong^®^ complex and clarithromycin (Clr) against *S. aureus*. (**A**)—time-kill curves of GoImmune Strong^®^ complex at different concentrations. (**B**)—Time-kill curves of combination of GoImmune Strong^®^ complex and clarithromycin. Each time-curve experiment was performed in duplicate and diluted samples were plated in duplicate on agar plates. Error bars indicate standard deviations.

A time-kill kinetic assay was executed using clarithromycin as the antibiotic that had synergy against all three bacteria in combination with the GoImmune Strong^®^ complex. Gram-positive *S. aureus* as the most susceptible and Gram-negative *K. pneumoniae* as the least susceptible bacteria were chosen for the test. The results of the time kill-kinetic assay of GoImmune Strong^®^ and antibiotic combinations confirmed the results obtained from checkerboard assays (Figure 1 and Figure 2).

GoImmune Strong^®^ alone at 2 MIC and 4 MIC concentrations reduced *K. pneumoniae* colony counts by 1.52 Log_10_ and 2.45 Log_10_ after 6 h. The reduction was statistically significant compared to control already after 3 h incubation (*p* < 0.001). No further reduction was observed over 24 h period. Clarithromycin did not reduce colony counts. Combination of GoImmune Strong^®^ and clarithromycin reduced colony counts already at 0.5 MIC concentrations within the first 6 h. Strong reduction by 1.6 Log_10_ and 3.84 Log_10_ was seen after 6 h incubation in presence of 2 MIC and 4 MIC concentrations. Reduction was statistically significant (*p* < 0.001) compared to control. When compared to clarithromycin alone, after 3 h incubation, colony count reduction in presence of the combination at 4 MIC concentrations was statistically significant (*p* < 0.001). After 6 h, the reduction in presence of antibiotic and GoImmune Strong^®^ combination was statistically significant compared to clarithromycin alone at all concentrations. Viable bacterial cells were not observed in presence of all concentrations of combination after 24 h period.

Clarithromycin did not have an effect on colony counts of *S. aureus.* GoImmune Strong^®^ complex alone did not reduce colony counts within the first 6 h of incubation. However, the differences in colony counts were statistically significant compared to control (*p* < 0.05). A statistically significant reduction by 1.28 Log was seen after 24 h for a 2 MIC concentration; in the presence of 4 MIC, no viable bacteria were detected. An effect of the combination of GoImmune Strong^®^ complex with the antibiotic was seen after 6 h—all tested concentrations reduced colony counts. The reduction in presence of antibiotic and GoImmune Strong^®^ combination was statistically significant (*p* < 0.001) compared to clarithromycin alone at all concentrations. After 24 h, a 1.96 Log_10_ reduction was observed in the presence of 1 MIC and 2 MIC concentrations; no viable bacteria were detected in the presence of a 4 MIC concentration of the combination.

## 3. Discussion

Natural compounds from various sources, including plants and bee products, have been characterized for various biological activities. Among them, antimicrobial activity is of particular interest in light of increasing antibiotic resistance and emerging new strains of pathogens. A promising approach is to use plant extracts or isolated compounds as boosters of antibiotic activity and tools for the restoration of susceptibility in resistant microorganisms [80,81]. The combination of various natural products and in-depth characterization of their biological activities is an important precondition for the successful repurposing of existing products for the prevention and treatment of infections.

In this study, a combination of commercially available plant and propolis extracts that is currently used as a food supplement, GoImmune Strong^®^, was tested for its antibacterial characteristics. GoImmune Strong^®^ was developed based on available data on antioxidative and immunity boosting properties of chosen plant extracts. Following market launch, observations and feedbacks from customers indicated on the potential positive effects of the product in respiratory infections. This urged to us to assess GoImmune Strong^®^ for its activity against selected respiratory pathogens and interaction with antibiotics to estimate its potential to be repurposed as a prophylactic and antimicrobial therapy-enhancing product. Two of the components of the GoImmune Strong^®^ complex, *O.europae* leaf extract and Brazilian green propolis extract, have been thoroughly studied for their biological activities. The third component, *T. avellanedae*, despite the wide traditional usage, is less characterized. To the best of our knowledge, there are no previous studies on biological activities of the combination of extracts of these three natural products.

Results showed that the complex of the extracts inhibits the growth of all tested microorganisms, with the most pronounced effect against *S. aureus*. This is in line with the published studies about individual components of the complex. Anti-staphylococcal activity is characteristic for all three extracts. Compared to some published studies, the MIC value of complex exceeds that of individual extracts, e.g., 2.68 mg/mL of olive leaf extract reported by Pereira et al. and 2.5 mg/mL by Karygianni et al., compared to 0.78 mg/mL in our study [21,23]; some other authors reported higher activity, e.g., a MIC value of 15.6 μg/mL of olive extract against MRSA [82], or similar results, such as MIC values for propolis in the range of 0.39–0.78 mg/mL [41]. In general, data about propolis and olive leaf extract activity against *S. aureus* vary widely among studies due to different collection sites and extraction methods [22,28,29,44,48,83]. The same applies to total phenolic content, where variations of concentrations mainly arise from different extraction methodologies.

*H. influenzae* and *K. pneumoniae* were less susceptible than Gram-positive *S. aureus*, a result that is similar to the effects of polyphenol-rich propolis extracts reported in the literature [44,84]. *Tabebuia* spp. are also reported to have lower or no activity against Gram-negative bacteria [54,55,57]. It is hypothesized that the high activity of the complex against *S. aureus* is due to the combined anti-staphylococcal effect of all three extracts, but the ability to inhibit the growth of Gram-negative *H. influenzae* and *K. pneumoniae* is due to the broader antimicrobial activity of olive leaf extract.

Natural extracts are complex mixtures of various compounds that possess multiple mechanisms of action. Synergistic activities between different extract components are possible. Some compounds identified in the components of GoImmune Strong^®^ are known for their activity against *S. aureus*, *K. pneumoniae* and *H. influenzae*. Hydroxytyrosol and oleuropein has been shown to be effective against all three bacteria [58,85,86,87]. Other broad spectrum compounds of GoImmune Strong^®^ that are effective against both Gram-positive and Gram-negative bacteria are cinnamic acid and its derivatives, verbascoside and isoverboscaside [88,89]. 4-hydroxybenzoic acid was detected in *T.avellanedae* bark extract and olive leaf extract and is well known for its activity against Gram-positive bacteria [90]. The modes of action of phenolic compounds are not yet fully elucidated. The variations in the activity against Gram-negative and Gram-positive bacteria might be explained by the differences in the cell surface. It has been proposed that the outer membrane of Gram-negative bacteria blocks penetration of antimicrobial compounds, making bacteria less susceptible [68]. Other studies point out that there is no clear correlation between Gram staining and susceptibility to phenolic compounds. Susceptibility varies between bacterial species or even strains and is dependent on the physico-chemical characteristics of the compounds [91].

A valuable finding of our study is the synergistic activity of the GoImmune Strong^®^ complex with antibiotics used to treat respiratory infections. Synergy against *H. influenzae* was detected for all tested antibiotics. Synergy with azithromycin and clarithromycin against *S. aureus* was observed. Against *K. pneumoniae,* the complex was synergistic only with clarithromycin. Synergy could be explained by the activity of individual extracts of the complex. In the literature, the synergy of propolis extracts with antibiotics acting on the bacterial cell wall and protein synthesis has been reported [41,43]. Olive leaf extracts have synergistic activities with beta-lactams [28]. The specific mechanisms of synergy and role of individual compounds is yet to be elucidated. It is hypothesized that synergistic activity arises from different targets and mechanisms of action of the components of the complex and antibiotics.

The results of our study show the potential of the GoImmune Strong^®^ complex to be used as an antibiotic booster, allowing for the enhancement of the efficacy of antibiotic therapy as well as a reduction in the administered concentrations of antibiotics for respiratory infections. At the same time, it is clear that further studies are needed to characterize the mechanisms of synergy and prove the antimicrobial activity in vivo. Tests in antibiotic-resistant microbial strains would be beneficial to further prove the applicability of the extract complex in the treatment of infections.

## 4. Materials and Methods

### 4.1. Extracts and Their Preparation for the Tests

Green propolis extract (BNatural, Corbetta, Italy), standardized to contain 5% total phenolic acids; *Tabebuia avellanedae* bark extract (EPO Instituto Farmochimico Fitoterapico, Milan, Italy), standardized to contain 3% *w*/*w* lapachol; and *Olea europaea* leaf extract (Gonmisol, Barcelona, Spain) standardized to contain 20% *w*/*w* oleuropein were combined in a ratio of 1:2.5:2, respectively. Ratio of the extracts was chosen to be identical to that of the commercial product GoImmune Strong^®^.

Prior to antimicrobial activity tests, complex was dissolved in dimethyl sulfoxide (Sigma-Aldrich, St. Louis, MO, USA) at concentration 200 mg/mL.

For total phenolic content analysis all plant extracts were dissolved in dimethyl sulfoxide (Sigma-Aldrich, St. Louis, MO, USA) at concentration 50 mg/mL; green propolis extract was dissolved in dimethyl sulfoxide at concentration 25 mg/mL.

### 4.2. Total Phenolic Content

Total phenolic content (TPC) was determined using Folin–Ciocalteu assay adjusted for microplates with gallic acid as the standard. Briefly, dilutions of extracts (25 μL) in 75 μL water were incubated with 25 μL 1N Folin–Ciocalteu reagent (Sigma-Aldrich, St. Louis, MO, USA) for 6 min at room temperature (22–24oC). A total of 100 μL 7% sodium carbonate was added to the reaction. The absorbance was measured at 760 nm using a microplate reader (TECAN Infinite 200PRO) after incubation for 90 min at room temperature in the dark. All samples were analyzed in duplicates, with three technical replicates. Results were expressed as mg of gallic acid equivalent (GAE) per g of dry weight (DW).

### 4.3. Chromatographic Analysis

#### 4.3.1. Preparation of Samples and Standards

The method of Ghomari et al., 2019 [58], was adapted for sample preparation of green propolis, *Tabebuia avellanedae* bark, and *Olea europaea* leaf extract. A total of 0.2 g of dried plant extracts was mixed with 2 mL of 80% (*v*/*v*) ethanol for 4 h. Samples were filtered through a nylon membrane filter (pore size 0.45 µm) and were injected into the HPLC system.

External standard calibration was used for quantitative determination of phenolic compounds and oleuropein. Analytical standards of oleuropein, chlorogenic acid, caffeic acid, p-coumaric acid, ferulic acid, naringenin, rutin, vanillic acid, cinnamic acid, 4-hydroxy benzoic acid, and protocatechuic acid were purchased from *Sigma-Aldrich*. Stock solutions of standards at a concentration of 1000 mg/L were prepared in mobile phase. Working solutions were prepared ranging from 0.5 to 25 mg/L by diluting the stock solutions with mobile phase. All stock and working solutions were stored at 4 °C temperature. The standard solution at each concentration was analyzed in triplicate. All calibration curves were constructed by plotting the average peak area against concentration.

#### 4.3.2. HPLC–TOF-HRMS Analysis

The chemical composition of the ethanolic extracts was determined using an *Agilent 1290 Infinity* series system (Agilent Technologies, Waldbronn, Germany) coupled to an *Agilent 6230 TOF LC/MS* (Agilent Technologies, Waldbronn, Germany) with electrospray ionization (ESI). Chromatographic separation of phenolic compounds and iridoids was performed at 30 °C using an Xterra MS C18, 2.1 × 150 mm, 3.5 μm column. The mobile phase consisted of aqueous 0.1% formic acid (A) and acetonitrile (B). The flow rate was 0.3 mL min^−1^, and gradient elution was performed according to the following program: 0 min, 2% B; 5.0 min, 2% B; 20.0 min, 95% B; 25.0 min, 95% B; 26.0 min, 2% B; 30.0 min, 2% B. The injection volume was 20 μL.

The mass spectrometry operating conditions were as follows in negative ionization mode: gas temperature 320 °C, gas flow rate 12 L/min, nebulizer pressure 40 psi, sheath gas temperature 320 °C, sheath gas flow 12 L/min, capillary voltage 4000 V, and applied fragmentor 130 V. The full scan mass range was set to 50–1500 *m*/*z*. Internal reference masses 112.98559 *m*/*z* and 1033.98811 *m*/*z* (G1969-85001 ES-TOF Reference Mass Solution Kit, Agilent Technologies & Supelco) were used. Spectral UV data from all peaks were accumulated in the range 200–600 nm, and chromatograms were monitored at 260, 280, and 520 nm. Spectrum extraction and peak detection were performed with MassHunter 7.00 Software (Agilent). Peak identification was determined by *m*/*z* values and standards.

### 4.4. Determination of MIC and MBC

Mueller–Hinton broth (Biolife, Milan, Italy) was used for susceptibility testing by twofold serial broth microdilution of *Staphylococcus aureus* MSCL 334 and *Klebsiella pneumoniae* MSCL 535 in aerobic conditions. Mueller–Hinton broth supplemented with yeast extract 5 mg/mL, hemin 15 µg/mL, and NAD 15 µg/mL was used for testing of *Haemophilus influenzae* MSCL 1619 in anaerobic conditions (GasPak Anaerobe Pouch, BD, USA).

A total of 10 mg/mL stock solution of antibiotics was prepared. Amoxiclav (Sandoz, Kundl, Austria; amoxicillin 875 mg, acid clavulanic 125 mg) was dissolved in sterile water. Azithromycin dihydrate (Sigma-Aldrich, St. Louis, MO, USA) was dissolved in ethanol and further diluted in water. Clarithromycin (Sigma-Aldrich, St. Louis, MO, USA) was dissolved in dimethyl sulfoxide (Sigma-Aldrich, St. Louis, MO, USA) and further diluted in water. Stock suspension of extract complex was prepared in dimethyl sulfoxide at concentration 200 mg/mL. Solutions of antibiotics and suspension of extract complex with different concentrations were freshly prepared on the day of the experiment.

The inoculum of bacteria was prepared in sterile water with density of 0.08–0.10 at A_625_ and diluted 100-fold in appropriate broth. Then, 96-well plates were incubated at 37 °C for 24 h. The MIC was determined as the lowest concentration of studied material, which showed no visible growth. From wells where growth was not detected, 4 μL of media was seeded on appropriate solidified media for MBC determination.

### 4.5. Determination of Fractional Inhibitory Concentrations (FIC) and FIC Index (FICI)

The combined effect of extract combination and antibiotics (FIC) was evaluated by modified microdilution chequerboard method (Fratini et al., 2017). Assay was performed on 96-well plate using previously determined MIC values. Seven concentrations of *the extract combination* were prepared (4 MIC, 2 MIC, MIC, 1/2 MIC, 1/4 MIC, 1/8 MIC, and 1/16 MIC). Dilutions of the extract combination were added on the x-axis across the chequerboard plate, while dilutions of antibiotic were dispensed on the y-axis in order to obtain six final concentrations (4 MIC, 2 MIC, MIC, 1/2 MIC, 1/4 MIC, and 1/8 MIC). The inoculum of bacteria was prepared in sterile water with density of 0.08–0.10 at A_625_ and diluted 100-fold in appropriate broth. Microplates were incubated at 37 °C for 24 h. FIC determinations were performed in triplicate.

FICI values were calculated using the following formula:

FICI = FIC*_antibiotic_* + FIC*_Complex_*

where

FIC*_antibiotic_* = MIC*_antibiotic_* in combination/MIC*_antibiotic_* alone

and

FIC*_Complex_* = MIC*_Complex_* in combination/MIC*_Complex_* alone.

Three different FICI interpretation methods were used to evaluate results. According to Fratini et al., 2017 [79], a synergistic effect (Syn_A_) is detected when FICI value < 1, a commutative effect (Com_A_) when FICI value = 1, an indifferent effect (Ind_A_) when 1 < FICI value ≤ 2, and an antagonistic effect (Ant_A_) when FICI value > 2.

According to Odds 2003 [78], a synergistic effect (Syn_O_) is observed when FICI value ≤ 0.5; an indifferent effect (Ind_O_) when 0.5 < FICI value ≤ 4, and an antagonistic effect (Ant_O_) when FICI value > 4.

According to EUCAST 2000 [77], a synergistic effect (Syn_E_) is observed when FICI value ≤ 0.5, an additive effect (Add_E_) when 0.5 < FICI value ≤ 1, an indifferent effect (Ind_E_) when 1 < FICI value < 2, and an antagonistic effect (Ant_E_) when FICI value ≥ 2.

### 4.6. Time-Kill Assay

The concentrations of half the MIC, equal to MIC, twice the MIC, and four times the MIC of the extract complex and antibiotics estimated in MIC assays were prepared. A bacterial inoculum with a final concentration of 10^6^ CFU/mL was added and incubated at 37 °C in an appropriate broth. A bacterial inoculum without added substances was used as a control. Aliquots of 0.1 mL in each tube were taken at time intervals of 0, 3, 6, and 24 h. Ten-fold serial dilutions were prepared and inoculated on appropriate solidified media and incubated at 37 °C for 24–48 h. The number of colony-forming units (CFU) was determined. A graph of the Log_10_ CFU/mL was plotted against time. Each time-curve experiment was performed in duplicate and diluted samples were plated in duplicate on agar plates. Error bars indicate standard deviations.

### 4.7. Data Analysis

Data were analyzed and graphs generated using GraphPad Prism 5.0 software (San Diego, CA, USA). Two-way ANOVA followed by multiple comparison test was used to test for differences between antibiotic combinations with GoImmune Strong^®^ and single concentrations over time. Differences were considered statistically significant if *p* ≤ 0.05.

## 5. Conclusions

In the era of antibiotic resistance, phenolic substances have become a subject of particular interest in prophylaxis and handling of bacterial infections. The testing of already available complexes of polyphenol-rich extracts revealed the antimicrobial activity against respiratory pathogens *S. aureus*, *K. pneumoniae*, and *H. influenzae*. The synergistic activity with beta-lactam and protein-synthesis-inhibiting antibiotics against both Gram-negative and Gram-positive bacteria points to the high potential of the complex to be applied in prophylaxis and treatment of respiratory infections. The mechanism of action and role of individual compounds in provision of the activity needs to be further investigated. The results of the study are an essential precondition for successful further repurposing of the polyphenol complex for use against bacterial infections. To achieve this, additional in vitro tests using multidrug-resistant strains are needed, followed by in vivo efficacy studies.

## Figures and Tables

**Table 1:** Total phenolic content of individual extracts and extract complex, gallic acid equivalents mg/g DW. TAE—*Tabebuia avellanedae* bark extract; OEE—*Olea europaea* leaf extract; GPE—green propolis extract. Mean ± SD from three independent analyses.

TAE	OEE	GPE	GoImmune Strong^®^
23.56 ± 3.37	171.69 ± 27.50	13.03 ± 2.12	63.37 ± 10.89

**Table 2:** Compounds identified in the *Olea europaea* leaf extract.

Compound	Formula	(M–H)^−^	RT, min	Refs.	Quantity Mean ± SD, μg/g DW
Hydroxytyrosol ^b,c^	C_8_H_10_O_3_	153.0557	7.12	[27,58]	1695 ± 11 ^d^
Hydroxytyrosol glucoside ^b,c^	C_14_H_20_O_8_	315.1085	7.78	[59]	203 ± 8 ^d^
Oleoside ^b,c^	C_16_H_22_O_11_	389.1089	7.94	[60]	44 ± 6 ^k^
4-Hydroxybenzoic acid ^a^	C_7_H_6_O_3_	137.0244	8.35	[61]	41 ± 3
Aesculin ^b,c^	C_15_H_16_O_9_	339.0722	8.59	[60]	21.8 ± 0.9 ^d^
Chlorogenic acid ^a^	C_16_H_18_O_9_	353.0878	9.01	[59]	17 ± 2
NI ^c^	–	377.1487	9.39	–	NQ
Luteolin rutinoside ^b,c^	C_27_H_30_O_16_	609.1461	9.61	[62]	5.7 ± 0.5 ^j^
C10 isoprenoid^c^	C_17_H_24_O_11_	403.1246	9.67	[63]	NQ
NI ^c^	–	305.0739	9.95		NQ
Demethyloleuropein ^b,c^	C_24_H_30_O_13_	525.1614	10.05	[63]	4.0 ± 0.2 ^k^
Hydroxyphenylacetic acid ^b,c^	C_8_H_8_O_3_	151.0401	10.09	[64]	0.858 ± 0.004 ^d^
Rutin ^a^	C_27_H_30_O_16_	609.1461	10.18	[21,27,58]	7.5 ± 0.2
Verbascoside ^b,c^	C_29_H_36_O_15_	623.1981	10.22	[21,27]	120.4 ± 0.6 ^e^
p-Coumaric acid ^a^	C_9_H_8_O_3_	163.0401	10.26	[58,61]	1.80 ± 0.04
Luteolin O-glucoside ^b,c^	C_21_H_20_O_11_	447.0933	10.41	[27,58,60]	31.0 ± 0.6 ^j^
Isoverbascoside ^b,c^	C_29_H_36_O_15_	623.1981	10.49	[27]	18.8 ± 1.3 ^e^
Ferulic acid ^a^	C_10_H_10_O_4_	193.0506	10.60	[58,61]	1.6 ± 0.2
Apigenin 7-O-glucoside ^b,c^	C_21_H_20_O_10_	431.0984	10.88	[21,27]	4.6 ± 0.4 ^j^
Luteolin O-glucoside ^b,c^	C_21_H_20_O_11_	447.0933	10.93	[21,27,60]	17.3 ± 1.1 ^j^
Elenolic acid ^b,c^	C_11_H_14_O_6_	241.0718	11.03	[59]	7.2 ± 0.4 ^k^
Oleuropein ^a^	C_25_H_32_O_13_	539.1770	11.10	[27,58,59,60]	8681 ± 85
NI ^c^	–	601.2146	11.61	–	NQ
Elenolic acid derivative ^b,c^	–	241.0718	12.16	–	2.5 ± 0.3 ^k^
Luteolin ^b,c^	C_15_H_10_O_6_	285.0405	12.20	[58,60]	18 ± 2 ^j^
Quercetin ^b,c^	C_15_H_10_O_7_	301.0354	12.22	[58]	21 ± 2 ^j^
NI ^c^	–	377.1253	12.47	–	NQ
Apigenin derivative ^b,c^	–	269.0455	12.96	–	1.1 ± 0.2^j^
NI ^c^	–	377.1255	13.23	–	208 ± 12 ^d^
NI ^c^	–	377.1255	13.45	–	53 ± 7 ^d^
Chrysoeriol ^b,c^	C_16_H_12_O_6_	299.0561	14.89	[65]	4.5 ± 0.2 ^j^
NI ^c^	–	545.1911	14.94	–	NQ
NI ^c^	–	721.3611	15.80	–	NQ
Apigenin derivative ^b,c^	–	269.0455	16.02	–	1.8 ± 0.3 ^j^
C30 isoprenoid ^c^	C_30_H_48_O_4_	471.3480	17.64	[63]	NQ
NI ^c^	–	401.3017	20.46	–	NQ
NI ^c^	–	401.3014	20.99	–	NQ

NI—not identified; NQ—not quantified; ^a^ confirmed by standard; ^b^ confirmed by reference; ^c^ confirmed by HRMS; ^d^ expressed as p-coumaric acid; ^e^ expressed as caffeic acid; ^j^ expressed as rutin; ^k^ expressed as oleuropein.

**Table 3:** Compounds identified in the *Tabebuia avellanedae* bark extract.

Compound	Formula	(M–H)^−^	RT, min	Refs.	Quantity Mean ± SD, μg/g DW
Protocatechuic acid derivative ^c^	–	153.0193 *	6.46	–	52 ± 7 ^l^
Protocatechuic acid ^a^	C_7_H_6_O_4_	153.0193	7.37	–	87 ± 9
2,4-dimethoxyphenyl 1-O-β-D-apiofuranosyl-(1–6)-β-D-glucopyranoside ^b,c^	C_19_H_28_O_12_	447.1509	7.95	[66]	5.61 ± 0.06 ^d^
4-Hydroxybenzoic acid ^a^	C_7_H_6_O_3_	137.0244	8.35	[54,57]	20.5 ± 0.5
Epiaucubin ^b,c^	C_15_H_22_O_9_	345.1191	8.41	[66]	3.0 ± 0.4 ^k^
NI ^c^	–	461.1284	8.49	–	121 ± 9 ^d^
NI ^c^	–	487.1431	8.55		NQ
C10 isoprenoids ^c^	C_19_H_28_O_11_	431.1559	8.67	[63]	NQ
Hydroxy benzoic acid isomer ^b,c^	C_7_H_6_O_3_	137.0244	8.70	[54]	521 ± 46 ^m^
Protocatechuic acid derivative ^c^	–	153.0193 *	8.73	–	9.4 ± 0.3 ^l^
NI ^c^	–	523.1660	9.00	–	NQ
Chlorogenic acid ^a^	C_16_H_18_O_9_	353.0878	9.05	[54]	0.49 ± 0.02
Protocatechuic acid derivative ^c^	–	153.0193 *	9.13	–	9.4 ± 0.3 ^l^
Specioside ^b,c^	C_24_H_28_O_12_	507.1508	9.16	[67]	2.6 ± 0.2 ^k^
10-O-(4-methoxybenzoyl)-impetiginoside A ^b,c^	C_23_H_28_O_12_	495.1508	9.37	[66]	3.1 ± 0.3 ^k^
Caffeic acid ^a^	C_9_H_8_O_4_	179.0350	9.39	[54]	6.87 ± 0.04
Flavone or flavonol ^c^	C_29_H_28_O_11_	551.1559	9.41	[63]	30 ± 4 ^j^
NI ^c^	–	639.1926	9.61	–	NQ
NI ^c^	–	521.1521	9.74	–	62 ± 6 ^d^
Calyxin ^c^	C_35_H_34_O_8_	581.2181	9.83	[68]	14.6 ± 0.4 ^i^
C15 isoprenoids ^c^	C_15_H_16_O_6_	291.0874	10.11	[68]	NQ
Verbascoside ^b,c^	C_29_H_36_O_15_	623.1976	10.22	[54]	355 ± 14 ^e^
p-coumaric acid ^a^	C_9_H_8_O_3_	163.0401	10.26	[54]	2.20 ± 0.04
1-benzyl-[6-p-hydroxybenzoyl]-b-D-glucopyranosyl-(1–3)-b-D-glucopyranoside ^b,c^ or 2-(4-hydroxyphenyl)ethy1, 1-O-β-D-[5-O-(4-hydroxybenzoyl)]-apiofuranosyl-(1–6)-β-D-glucopyranoside ^b,c^	C_26_H_32_O_13_	551.1770	10.40	[66,69]	26.0 ± 1.1 ^d^
3,4-dimethoxyphenyl 1-O-β-D-[5-O-(4- methoxybenzoyl)]-apiofuranosyl-(1–6)-β-D-glucopyranoside ^b,c^	C_27_H_34_O_14_	581.1875	10.43	[66]	2.11 ± 0.09 ^d^
Astragalin ^b,c^	C_21_H_20_O_11_	447.0933	10.46	[69]	15.1 ± 0.8 ^j^
5,7-Dihydroxy-3’,4’-dimethoxyflavanone 7-rutinoside ^c^	C_29_H_36_O_15_	623.1981	10.50	[63]	265 ± 20 ^i^
Isoverbascoside ^b,c^	C_29_H_36_O_15_	623.1976	10.53	[54]	279 ± 3 ^e^
6-O-(4-methoxybenzoyl)-5,7-bisdeoxycynanchoside ^b,c^	C_23_H_30_O_12_	497.1664	10.56	[66]	5.8 ± 0.3 ^k^
Veratic acid ^b,c^	C_9_H_10_O_4_	181.0506	10.64	[54,57]	68 ± 7 ^n^
C15 isoprenoid ^c^	C_15_H_16_O_6_	291.0857	10.74	–	NQ
Phenolic compound glycoside ^b,c^	C_27_H_34_O_15_	597.1825	10.81	[70]	24 ± 3
Veratic acid derivative ^c^	–	181.0506 *	11.02	–	15 ± 3 ^n^
2-(4-hydroxyphenyl)ethyl, 1-O-β-D-[5-O-(3,4-dimethoxybenzoyl)]-apiofuranosyl-(1–6)-β-D-glucopyranoside ^b,c^	C_28_H_36_O_14_	595.2031	11.04	[66]	70.3 ± 1.2 ^d^
NI ^c^	–	539.1779	11.15	–	62 ± 2 ^d^
Quercetin 5,7,3’,4’-tetramethyl ether Quercetin 5,7,3’,4’-tetramethyl ether 3-rutinoside ^c^	C_31_H_38_O_16_	665.2087	11.27	[63]	230 ± 19 ^j^
2-(4-hydroxyphenyl)ethyl 1-O-β-D-[5-O-(4 methoxybenzoyl)]-apiofuranosyl-(1–6)-β-D-glucopyranoside ^b,c^	C_27_H_34_O_13_	565.1926	11.49	[66]	11.8 ± 1.5 ^d^
NI ^c^	–	527.1768	11.55	–	74.1 ± 1.4 ^d^
Ferulic acid derivative ^c^	–	193.0506 *	11.92	–	112 ± 15 ^h^
Kaempferol deivative ^c^	–	285.0405 *	12.24	–	3.0 ± 0.3 ^j^
NI ^c^	–	327.2181	12.70	–	NQ
NI ^c^	–	329.2345	13.14	–	NQ
5-hydroxy-2-(1-hydroxyethyl)naphtho [2,3-b]furan-4,9-dione or 8-hydroxy-2-(1-hydroxyethyl)naphtho [2,3-b]furan-4,9-dione ^b,c^	C_14_H_10_O_5_	257.0455	13.37	[71,72]	NQ
Alkyl hydroquinone or derivative ^c^	C_17_H_26_O_4_	293.1758	14.72	[63]	NQ
NI ^c^	–	385.2556	16.19	–	NQ
NI ^c^	–	285.2042	16.42	–	NQ
NI ^c^	–	311.2196	17.02	–	NQ
NI ^c^	–	327.1776	17.14	–	NQ

NI—not identified; NQ—not quantified; *—MS fragments; ^a^ confirmed by standard; ^b^ confirmed by reference; ^c^ confirmed by HRMS; ^d^ expressed as p-coumaric acid; ^e^ expressed as caffeic acid; ^h^ expressed as ferulic acid; ^i^ expressed as naringenin; ^j^ expressed as rutin; ^k^ expressed as oleuropein; ^l^ expressed as protocatechuic acid; ^m^ expressed as p-hydroxy benzoic acid; ^n^ expressed as vanillic acid.

**Table 4:** Compounds identified in the green propolis extract.

Compound	Formula	(M–H)^−^	RT, min	Refs.	Quantity Mean ± SD, μg/g DW
Chlorogenic acid isomer ^b,c^	C_16_H_18_O_9_	353.0878	8.25	[73,74,75]	15 ± 2 ^f^
Chlorogenic acid ^a^	C_16_H_18_O_9_	353.0878	9.05	[50,74]	105 ± 4
Caffeic acid ^a^	C_9_H_8_O_4_	179.0350	9.39	[73,76]	45 ± 6
Flavone or flavonol ^c^	C_25_H_28_O_12_	519.1508	9.97	[63]	650 ± 33 ^d^
p-coumaric acid ^a^	C_9_H_8_O_3_	163.0401	10.26	[73,74,76]	256 ± 23
Ferulic acid ^a^	C_10_H_10_O_4_	193.0506	10.52	[73,76]	8.1 ± 0.6
Dicaffeoylquinic acid isomer ^b,c^	C_25_H_24_O_12_	515.1195	10.66	[50,73,74,75]	97 ± 9 ^e^
Dicaffeoylquinic acid isomer^b,c^	C_25_H_24_O_12_	515.1195	10.81	[50,73,74,75]	97±6^e^
Dicaffeoylquinic acid isomer ^b,c^	C_25_H_24_O_12_	515.1195	11.00	[50,73,74,75]	321 ± 27 ^e^
Caffeic acid derivative ^b,c^	–	487.1563	11.39	[73]	32.9 ± 0.8 ^e^
Luteolin methyl ether crotonylglucoside or luteolin glucoside methyl butanoate ^c^	C_26_H_26_O_12_	529.1315	11.61	[63]	17.1 ± 1.2 ^j^
Caffeic acid prenyl ester ^b,c^	C_14_H_16_O_4_	247.0976	11.83	[76]	18.4 ± 1.3 ^e^
Tricaffeoylquinic acid ^b,c^	C_34_H_30_O_15_	677.1512	12.02	[73]	98 ± 9 ^e^
Dimethyl-dicaffeoylquinic acid ^b,c^	C_27_H_28_O_12_	543.1508	12.13	[73]	40 ± 4 ^e^
Dimethyl-dicaffeoylquinic acid ^b,c^	C_27_H_28_O_12_	543.1508	12.36	[73]	46 ± 6 ^e^
3,4-Dimethyl caffeic acid	C_11_H_12_O_4_	207.0663	12.43	[76]	13.6 ± 1.0 ^e^
Chlorogenic acid derivative ^c^	–	353.0878 *	12.55	–	4.2 ± 0.2 ^f^
Chlorogenic acid derivative ^c^	–	353.0878 *	12.71	–	3.3 ± 0.2 ^f^
Naringenin ^a^	C_15_H_12_O_5_	271.0612	12.96	[73]	27 ± 2
Hesperetin ^b,c^	C_16_H_14_O_6_	301.0718	13.04	[73]	270 ± 16 ^i^
Kaempferol ^b,c^	C_15_H_10_O_6_	285.0405	13.10	[73,76]	17 ± 2^j^
Isorhamnetin ^b,c^	C_16_H_12_O_7_	315.0510	13.14	[50,73,76]	21 ± 3 ^j^
Caffeic acid derivative ^b,c^	–	705.1835	13.31	[73]	16 ± 2 ^e^
NI ^b,c^	–	301.1495	13.47	[73]	25 ± 2 ^d^
Rhamnetin ^b,c^	C_16_H_12_O_7_	315.0510	13.56	[50,73]	25 ± 3 ^j^
3,4-Dihydroxy 5-prenylcinnamic acid ^b,c^	C_14_H_16_O_4_	247.0976	13.61	[50,73,74,75]	121 ± 8 ^g^
NI ^b,c^	–	331.1613	13.72	[73]	140 ± 6 ^d^
Coumaric acid prenyl ester ^b,c^	C_14_H_16_O_3_	231.1027	13.99	[73,76]	301 ± 11 ^d^
4-Hydroxy-3-prenylcinnamic acid ^b,c^	C_14_H_16_O_3_	231.1027	13.99	[50,73,74,75]	327 ± 24 ^g^
Caffeic acid benzyl ester ^b,c^	C_16_H_14_O_4_	269.0819	14.45	[73,76]	3.0 ± 0.3 ^e^
Coumaric acid derivative ^b,c^	–	315.1601	14.50	[73]	56 ± 7 ^d^
Kaempferide derivative ^b,c^	–	377.1957	14.60	[73]	36.5 ± 1.3 ^j^
Sakuranetin ^b,c^	C_16_H_14_O_5_	285.0768	14.63	[73]	23.3 ± 1.5 ^i^
Chrysin ^b,c^	C_15_H_10_O_4_	253.0506	14.65	[50,76]	10.1 ± 0.9 ^j^
Pinocembrin ^b,c^	C_15_H_12_O_4_	255.0663	14.68	[50,73,76]	10.1 ± 0.7 ^i^
Kaempferide derivative ^b,c^	–	377.1953	14.75	[73]	59 ± 3 ^j^
Galangin ^b,c^	C_15_H_10_O_5_	269.0455	14.80	[50,76]	7.3 ± 0.5 ^j^
Caffeic acid phenethyl ester ^b,c^	C_17_H_16_O_4_	283.0976	14.83	[50,73,76]	7.4 ± 0.2 ^e^
Kaempferide ^b,c^	C_16_H_12_O_6_	299.0561	14.89	[50,73,76]	110 ± 11 ^j^
Dimethoxyquercetin ^b,c^	C_17_H_14_O_7_	329.0667	15.01	[50]	33.6 ± 0.5 ^j^
Dicoumaric prenyl ester ^b,c^	C_23_H_22_O_6_	393.1344	15.08	[73,76]	50 ± 6 ^d^
Caffeic acid cinnamyl ester ^b,c^	C_18_H_16_O_4_	295.0976	15.35	[76]	6.5 ± 0.2 ^d^
Kaempferide derivative ^b,c^	–	529.1497	15.38	[73]	22.4 ± 0.2 ^j^
Coumaric acid derivative ^b,c^	–	315.1600	15.44	[73]	52 ± 5 ^d^
Baccharin ^b,c^	C_29_H_38_O_11_	561.2341	15.61	[50,74]	1.5 ± 0.2 ^d^
Artepillin C derivative ^b,c^	–	329.1780	15.72	[73]	156 ± 24 ^d^
Coumaric acid derivative ^b,c^	–	559.1628	15.78	[73]	1.81 ± 0.02 ^d^
Artepillin C ^b,c^	C_19_H_24_O_3_	299.1653	16.39	[50,73,74,75]	414 ± 26 ^d^
NI ^c^	–	613.2155	16.72	–	NQ
NI ^c^	–	613.3219	17.50	–	NQ
NI ^c^	–	627.2288	17.66	–	NQ
C20 isoprenoid ^c^	C_20_H_30_O_2_	301.2226	19.14	[63]	NQ

NI—not identified; NQ—not quantified; *—MS fragments; ^a^ confirmed by standard; ^b^ confirmed by reference; ^c^ confirmed by HRMS; ^d^ expressed as p-coumaric acid; ^e^ expressed as caffeic acid; ^f^ expressed as chlorogenic acid; ^g^ expressed as cinnamic acid; ^i^ expressed as naringenin; ^j^ expressed as rutin.

**Table 5:** Minimal inhibitory (MIC) and minimal bactericidal (MBC) concentrations GoImmune Strong^®^ complex and selected antibiotics.

	*S. aureus*		*K. pneumoniae*		*H. influenzae*	
	MIC	MBC	MIC	MBC	MIC	MBC
Azithromycin (μg/mL)	0.5	>128	8	>128	0.25	>128
Clarithromycin (μg/mL)	0.25	128	64	>128	0.5	>128
Amoxiclav (μg/mL)	0.13	1.0	8.0	64	8	>128
GoImmune Strong^®^ (mg/mL)	0.78	1.56	12.5	12.5	3.13	12.5

**Table 6:** Fractional inhibitory concentrations (FIC), FIC indexes, and interpretations of them. FIC_antib._—fractional inhibitory concentration of antibiotic; FIC_Comp._—fractional inhibitory concentration of the GoImmune Strong^®^ complex.

Antibiotic		Azithromycin	Clarithromycin	Amoxiclav
***Staphylococcus aureus***	FIC_antib._	0.25	0.06	0.5
	FIC_Comp_	0.06	0.03	0.5
	**FICI**	0.31	0.09	1.0
		Synergy ^a,b,c^	Synergy ^a,b,c^	Additive ^a^, commutative ^c^; no interaction ^b^
***Klebsiella pneumoniae***	FIC_antib._	1.0	0.5	0.5
	FIC_Comp_	0.5	0.03	0.5
	**FICI**	1.5	0.53	1.0
		No interaction ^b,c^	Additive ^a^; no interaction ^b^; synergy ^c^	Additive ^a^, commutative ^c^; no interaction ^b^
***Haemophilus influenzae***	FIC_antib._	0.12	0.25	0.13
	FIC_Comp_	0.25	0.13	0.25
	**FICI**	0.37	0.38	0.38
		Synergy ^a,b,c^	Synergy ^a,b,c^	Synergy ^a,b,c^

Methods used for interpretation: ^a^ EUCAST, 2000 [77]; ^b^ Odds, 2003 [78]; ^c^ Fratini et al., 2017 [79].

## Data Availability

Not applicable.
